# Gene Expression Analysis of Biphasic Pleural Mesothelioma: New Potential Diagnostic and Prognostic Markers

**DOI:** 10.3390/diagnostics12030674

**Published:** 2022-03-10

**Authors:** Rossella Bruno, Anello Marcello Poma, Greta Alì, Claudia Distefano, Agnese Proietti, Antonio Chella, Marco Lucchi, Franca Melfi, Renato Franco, Gabriella Fontanini

**Affiliations:** 1 Unit of Pathological Anatomy, University Hospital of Pisa, 56100 Pisa, Italy; rossella.bruno@for.unipi.it (R.B.); greta.ali@gmail.com (G.A.); distefano.claudia@hotmail.it (C.D.); agneseproietti@gmail.com (A.P.); 2 Department of Surgical, Medical, Molecular Pathology and Critical Area, University of Pisa, 56100 Pisa, Italy; marcellopoma@gmail.com; 3 Unit of Pneumology, University Hospital of Pisa, 56100 Pisa, Italy; anto.kell@tiscali.it; 4 Division of Thoracic Surgery, Department of Surgical, Medical and Molecular Pathology and Critical Care Medicine, University of Pisa, 56100 Pisa, Italy; marco.lucchi@unipi.it; 5 Minimally Invasive and Robotic Thoracic Surgery, Robotic Multispecialty Center of Surgery, University Hospital of Pisa, 56100 Pisa, Italy; franca.melfi@unipi.it; 6 Pathology Unit, Department of Mental and Physical Health and Preventive Medicine, Università degli Studi della Campania “L Vanvitelli”, 80138 Naples, Italy; renato.franco@unicampania.it

**Keywords:** biphasic pleural mesothelioma, gene expression, nanoString system, diagnosis, prognosis, biomarkers

## Abstract

Biphasic is the second most common histotype of pleural mesothelioma (PM). It shares epithelioid and sarcomatoid features and is challenging to diagnose. The aim of this study was to identify biphasic PM markers to improve subtyping and prognosis definition. The expression levels of 117 cancer genes, evaluated using the nanoString system, were compared between the three major histotypes (epithelioid, sarcomatoid, and biphasic), and expression differences within biphasic PM were evaluated in relation to the percentage of epithelioid components. Biphasic PM overexpressed *CTNNA1* and *TIMP3* in comparison to sarcomatoid, and *COL16A1* and *SDC1* in comparison to epithelioid PM. *CFB*, *MSLN*, *CLDN15*, *SERPINE1*, and *PAK4* were deregulated among all histotypes, leading to the hypothesis of a gradual expression from epithelioid to sarcomatoid PM. According to gene expression, biphasic PM samples were divided in two clusters with a significant difference in the epithelioid component. *ADCY4*, *COL1A1*, and *COL4A2* were overexpressed in the biphasic group with a low percentage of epithelioid component. Survival analysis using TCGA data showed that high *COL1A1* and *COL4A2* expression levels correlate with poor survival in PM patients. Herein, we identified markers with the potential to improve diagnosis and prognostic stratification of biphasic PM, which is still an orphan tumor.

## 1. Introduction

Pleural mesothelioma (PM) is a rare and highly aggressive tumor, mostly associated with asbestos exposure [1,2]. PM is characterized by a long latency period (10–30 years) from asbestos exposure to the clinical onset of disease, with a median overall survival ranging from 12–18 months and a 5-year survival rate of about 5% [3,4]. According to the 5th World Health Organization (WHO) Thoracic Tumors classification, diffuse PM includes three major histotypes: epithelioid (70–80% of cases), sarcomatoid (10%), and biphasic or mixed (10–20%) [5]. An accurate histological classification is mandatory to inform treatment decision and constitutes the most reliable prognostic factor. In fact, the reported median overall survival rate for epithelioid, biphasic and sarcomatoid PM is 19, 12, and 4 months, respectively [6,7]. Patients with epithelioid mesotheliomas can be eligible for surgery and multimodality approach, while sarcomatoid tumors do not achieve benefit from surgery [1]. The management of patients suffering from biphasic mesothelioma is challenging since this histotype displays a large degree of heterogeneity. A PM is classified as biphasic if both epithelioid and sarcomatoid components are present, with each comprising at least 10% of the tumor, and the proportion of sarcomatoid component is the major prognostic indicator and treatment driver [8,9]. Currently, there is no recommendation on grading sarcomatoid features, and percentage cut-offs of sarcomatoid component clearly associated with poor prognosis have not been identified [8]. Furthermore, the identification of a spindle cell component as malignant is not easy, and a low inter-observer correlation has been reported in recognizing biphasic PM [8]. In addition, the histological classification of PM is even more difficult on small biopsies, with a concordance with surgical resection ranging from 72 to 83% [10]. Recently, Chirieac and collaborators compared pre-surgery biopsies and surgical resection specimens from 759 PM patients: 112 patients (19%), initially diagnosed as epithelioid, were reclassified as biphasic after surgical resection; 15 (11%) and 4 (3%) cases initially diagnosed as biphasic were reclassified as epithelioid and sarcomatoid, respectively; 7 (19%) cases initially diagnosed as sarcomatoid were reclassified as biphasic [10].

The identification of histotype biomarkers to refine the histological classification and patients’ risk stratification is warranted. In this context, gene expression analysis proved to be a useful approach to better subtype PM [11]. Bueno and collaborators, by performing a transcriptome analysis, identified four distinct molecular subtypes: sarcomatoid, epithelioid, biphasic–sarcomatoid and biphasic–epithelioid, with the epithelioid subtype showing the best overall survival. The molecular subtyping was highly concordant with the histological diagnosis for sarcomatoid PM. A total of 62% of cases histologically diagnosed as epithelioid were differently reclassified, mostly as biphasic–epithelioid cases. A total of 21% of histologically biphasic samples were reclassified as sarcomatoid, 33.8% as biphasic–epitheliod and 40% as biphasic–sarcomatoid [11]. In the same way, Alcala and collaborators, using RNA-seq data, found that molecular profiles explain the prognosis of PM better than histological classification, with significant differences in the expression of proangiogenic and immune checkpoint genes [12].

The aim of our study was to identify histotype-specific biomarkers by evaluating the expression levels of 117 cancer genes, previously reported as deregulated in PM [13]. We mainly focused on biphasic PM, in order to underline differences with epithelioid and sarcomatoid PM and to investigate new potential prognostic biomarkers correlated with the amount of epithelioid component.

## 2. Materials and Methods

### 2.1. Study Population

In this study, PM samples (biopsies and surgical resections) were retrospectively collected from the archives of the Pathology Unit, University Hospital of Pisa (Pisa, Italy) and of the Pathology Unit, “Luigi Vanvitelli” University Hospital of Naples (Naples, Italy). The study was conducted in accordance with the principles of Helsinki declaration of 1975 and approved by our institutional ethics committee (protocol code 9989, 2019). Informed written consent for publication was not required because no sensitive data that could identify patients were used in this study.

**Figure 1:**
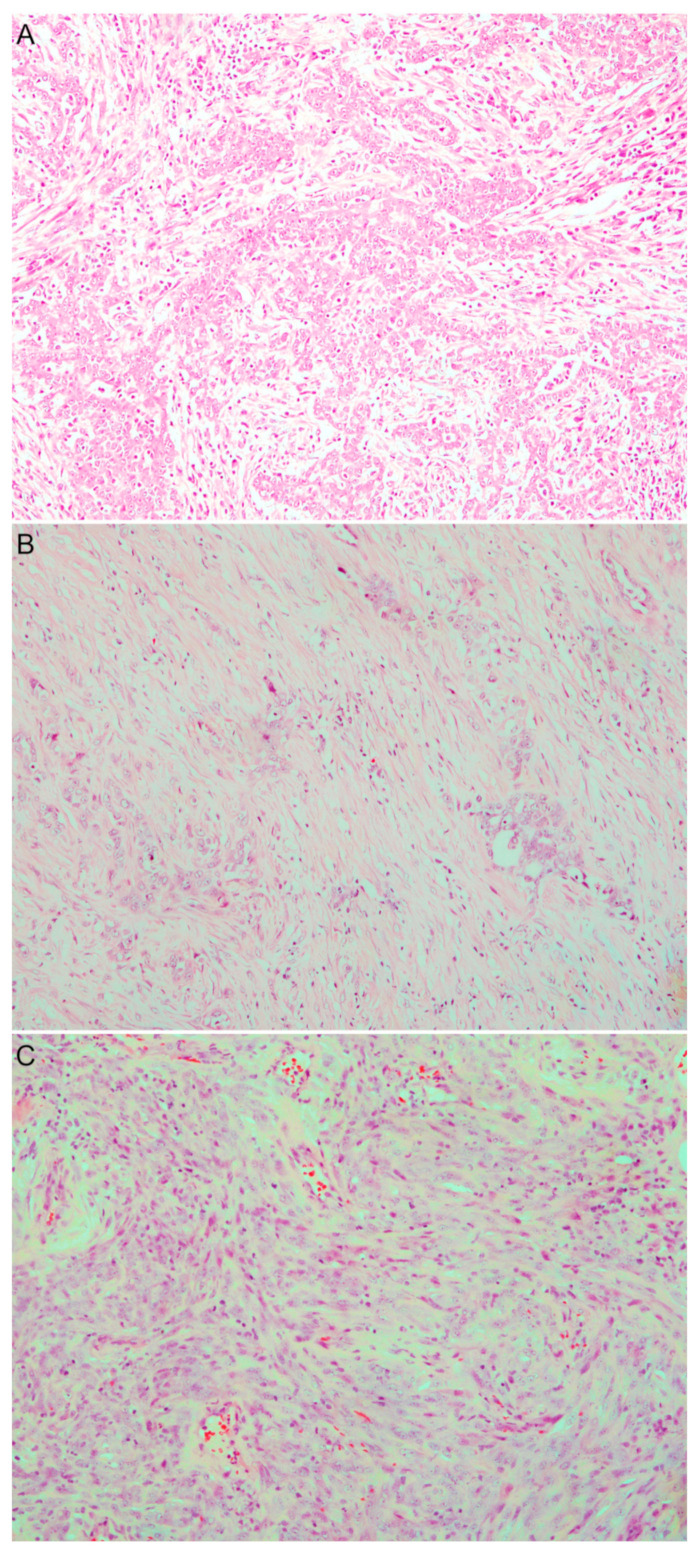
Histological images of diffuse pleural mesothelioma. (**A**) Epithelioid mesothelioma characterized by a tubular and solid pattern; (**B**) biphasic mesothelioma showing both epithelioid and sarcomatoid malignant areas; (**C**) sarcomatoid mesothelioma characterized by malignant spindle cells within a fibrous stroma. (Hematoxylin and Eosin staining, magnification 10×).

In detail, 25 biphasic (13 biopsies and 12 surgical resections), 32 epithelioid (10 biopsies and 22 surgical resections) and 18 sarcomatoid (11 biopsies and 7 surgical resections) samples were analyzed. All tumor samples were formalin-fixed and paraffin-embedded (FFPE); histological diagnosis and pathological features were independently reviewed by two pathologists (GA and GF) according to the WHO 5th edition criteria [5] (Figure 1). In particular, epithelioid mesotheliomas are usually composed of round, epithelioid cells, typically displaying eosinophilic cytoplasm and round nuclei with small nucleoli, although cytological atypia can occur. Epithelioid mesothelioma displays a wide range of histological patterns; common patterns are tubulopapillary, solid and trabecular, while adenomatoid is less common. Sarcomatoid mesotheliomas are instead composed of elongated/spindle cells arranged in solid sheets or within a fibrous stroma. Biphasic mesothelioma is a highly characteristic pattern containing a mixture of epithelioid and sarcomatoid areas within the same tumor. According to WHO criteria, in definitive resection specimens each pattern should constitute at least 10% of the neoplasm, while in small biopsy specimens any tumor can be diagnosed as biphasic PM, regardless of percentages of each component. However, reporting the percentage of sarcomatoid component is recommended because of potential implications for prognosis and therapeutic management. In the present study, for biphasic PM the percentage of epithelioid component was independently determined by two pathologists. All cases were discussed and the median value was considered for statistical evaluation. Since sarcomatoid areas may sometimes be difficult to distinguish from reactive stroma, we evaluated the fibrous proliferation using immunohistochemistry tests. In detail, immunohistochemical expression of mesothelial markers (i.e., calretinin, WT1, D2-40, and CK-PAN) or BAP-1 loss were helpful for diagnosis. Finally, the most representative paraffin block of each tumor was selected for gene expression analysis.

### 2.2. Gene Expression Analysis

Gene expression analysis was performed using a previously defined nCounter custom codeset including 117 PM target genes and 6 housekeeping genes, synthesized by nanoString Technologies (NanoString Technologies, Seattle, WA, USA), as previously described [13]. The 117 genes included in the panel belong to pathways with a crucial role in cancer development and progression (i.e., epithelial to mesenchimal transition (EMT), cell matrix, angiogenesis, DNA methylation, focal adhesion) [13].

For each sample, three FFPE unstained sections underwent a standard deparaffinization procedure; enrichment of tumor cells was performed by manual macrodissection, and RNA was purified using the Qiagen RNeasy FFPE kit (Qiagen, Hilden, Germany) according to the manufacturers’ instructions.

Quantity and quality of total RNA were assessed by a Xpose spectrophotometer (Trinean, Gentbrugge, Belgium). A total of 150 ng of RNA was used for nanoString analysis; hybridization was performed for 18 h at 65 °C in a SensoQuest thermal cycler (SensoQuest, Gottingen, Germany). The clean-up of samples and counts of digital reports were executed as described by the manufacturer (NanoString Technologies).

### 2.3. Data Analysis

Raw data were normalized using the nSolver v.4.0 (NanoString Technologies), and batch effects were removed by using the ComBat function of the Surrogate Variable Analysis (sva) Bioconductor package v.3.42.0. To observe gross similarity patterns of expression, hierarchical clustering and principal component analysis were performed using the packages heatmap3 v.1.1.9 and factoextra v.1.0.7 respectively. Differentially expressed genes (DEG) were computed by the moderate t-statistics and the Benjamini–Hochberg correction following the procedures of the limma package v.3.50.0. A false discovery rate (FDR) of 0.05 was considered significant. Common deregulated genes among contrasts were presented using a Venn diagram. To highlight gene expression differences within biphasic PM, non-negative matrix factorization algorithm was applied comparing the results with a random expression matrix. The best fitting clustering was used to predict the biphasic PM group based on gene expression; the analyses were performed using the NMF package v.0.23.0. The Mann–Whitney U test was used for testing differences according to the epithelial component.

For validation, TCGA data on Mesothelioma [11] were downloaded from cBioPortal (https://www.cbioportal.org/, last accessed on 10 December 2021) [14,15]. Patients were dichotomized based on median expression value of the selected genes. Survival curves were built using the Kaplan–Meier method and compared by the log-rank test following the procedures of the survival v.3.2-13 and survminer v.0.4.9 packages. All analyses and plots were generated in R v.4.1.2 environment (https://www.r-project.org/, last accessed on 15 December 2021).

## 3. Results

### 3.1. Differentially Expressed Genes between Histotypes

All RNA samples were adequate for gene expression analysis and none failed the nanoString test.

**Figure 2:**
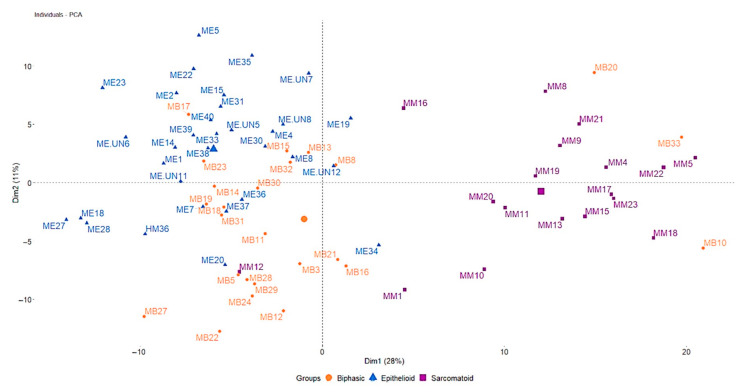
Principal component analysis. Epithelioid (blue) and sarcomatoid (magenta) PM are clearly separated, biphasic PM seems more heterogeneous and overlaps with the two other histotypes, especially epitheliod. MB, biphasic pleural mesothelioma; ME, epithelioid pleural mesothelioma; MM, sarcomatoid pleural mesothelioma.

**Figure 3:**
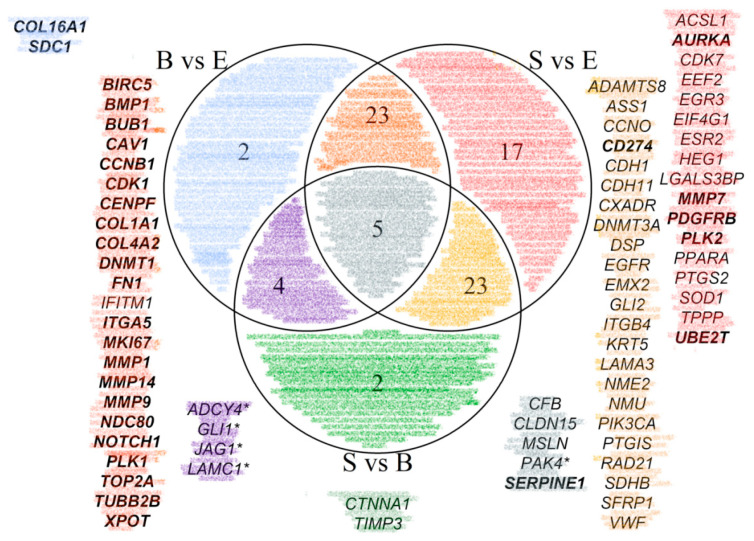
Venn diagram. Overlapping of differentially expressed genes among epithelioid, biphasic and sarcomatoid PM. Color codes identify subgroups of deregulated genes. In bold are genes, consistently upregulated in histotypes with higher sarcomatoid component, while plain font is for genes consistently upregulated in histotypes with higher epithelioid component. Asterisks identify genes that are highly expressed in biphasic compared to both epithelioid and sarcomatoid tumors. B, biphasic pleural mesothelioma; E, epithelioid pleural mesothelioma; S, sarcomatoid pleural mesothelioma.

**Figure 4:**
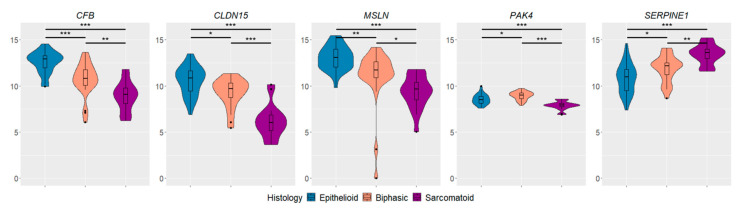
Violin plots. Genes deregulated among the three PM subtypes. * FDR < 0.05; ** FDR < 0.01; *** FDR < 0.001.

Principal component analysis indicated the existence of specific expression patterns for epithelioid and sarcomatoid PM, whereas biphasic PM showed a partial overlap with the other histotypes (Figure 2). The analysis of differentially expressed genes revealed 34 genes deregulated between biphasic and epithelioid PM, 68 between sarcomatoid and epithelioid PM, and 34 between sarcomatoid and biphasic PM (Tables S1–S3; Figures S1–S3). The overlapping of deregulated genes among contrasts is reported in the Venn diagram (Figure 3). Only four genes were specifically up-regulated in biphasic in comparison to sarcomatoid (*CTNNA1* and *TIMP3*) and epithelioid PM (*COL16A1* and *SDC1*). Twelve genes were down-regulated only in sarcomatoid in comparison to epithelioid PM (*ACSL1*, *CDK7*, *EEF2*, *EGR3, EIF4G1*, *ESR2*, *HEG1*, *LGALS3BP*, *PPARA*, *PTGS2*, *SOD1*, and *TPPP*) and 5 were up-regulated only in sarcomatoid compared to epithelioid PM (*AURKA*, *MMP7*, *PDGFRB*, *PLK2*, *UBE2T*). Five out of one-hundred-and-seventeen genes were deregulated among all groups (*CFB*, *MSLN*, *CLDN15*, *SERPINE1*, and *PAK4)* (Figure 4).

### 3.2. Gene Expression Differences within Biphasic PM

**Figure 5:**
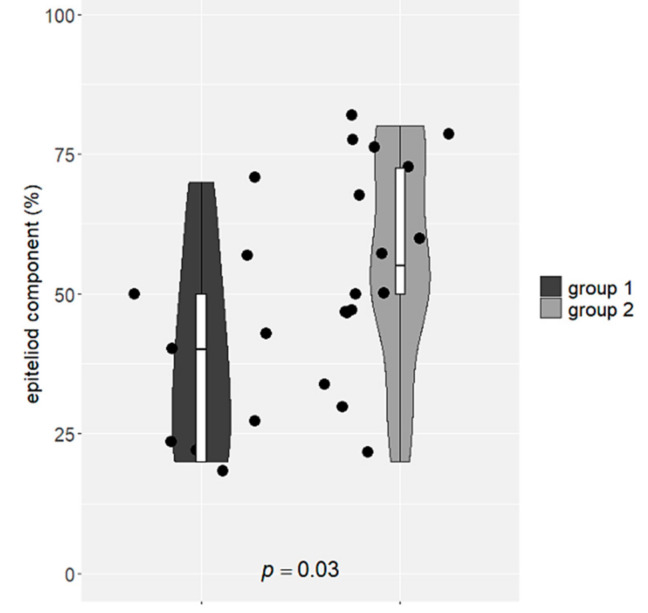
Violin plot of epithelial component according to biphasic groups identified by the non-negative matrix factorization algorithm on the basis of gene expression levels. The percentage of epithelioid component was separately and independently determined and it was statistically different among the two identified clusters. Median percentage of epithelioid component in group 1: 40% (interquartile range: 20–52.5%). Median percentage of epithelioid component in group 2: 55% (interquartile range: 50–72.5%).

**Figure 6:**
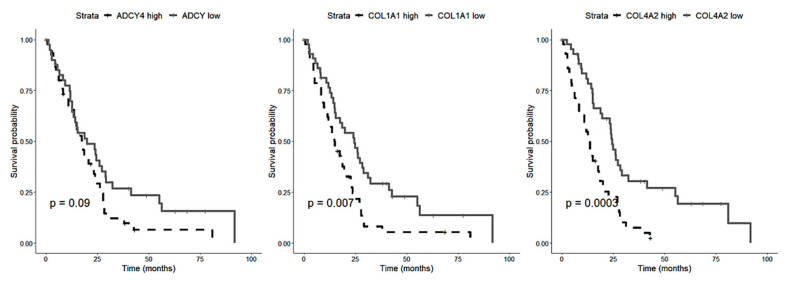
Overall survival curves of mesothelioma cases from *TCGA* cohort.

The best fitting clustering by the non-negative matrix factorization algorithm (Figures S4 and S5) predicted two biphasic PM groups based on gene expression. The percentage of the epithelioid component, independently evaluated, was significantly different between the two identified biphasic PM clusters, *p* = 0.03 (Figure 5). The comparison of gene expression levels between the two identified biphasic PM clusters revealed three genes significantly up-regulated in the cluster with a low percentage of epithelioid component: *ADCY4*, *COL1A1*, and *COL4A2*. Survival analysis using TCGA data showed that high *COL1A1* and *COL4A2* expression levels are correlated with a worse overall survival in PM patients (Figure 6).

## 4. Discussion

The histological subtyping of diffuse pleural mesothelioma is still the major prognostic factor, which is crucial for treatment planning [16]. Patients with epithelioid PM can be eligible for surgery, while those with sarcomatoid PM do not benefit from surgery [3,17]. Since biphasic PM shares both epithelioid and sarcomatoid features, it is hard both to diagnose and to treat [8,18]. Several studies have pointed out the diagnostic difficulties related to the pathological identification of biphasic PM, and the determination of sarcomatoid and epithelioid components, which greatly impact prognosis [8,10,18]. However, only few studies have been specifically focused on this histotype, mainly due to its rarity, and the identification of diagnostic and prognostic biomarkers is urgently needed.

Herein, we compared expression levels of 117 genes with a key role in cancer development and progression between the three major PM histotypes. As expected, expression levels mainly differ between sarcomatoid and epithelioid PM. Biphasic PM showed a heterogeneous gene expression profile, which overlaps to that of epitheliod PM in the majority of cases. Only four genes were specifically up-regulated in biphasic in comparison to sarcomatoid (*CTNNA1* and *TIMP3)* and epithelioid PM (*COL16A1* and *SDC1).* These deregulated genes impact on EMT process, leading from epithelial to mesenchymal phenotype [19]. In particular, *CTNNA1* and *TIMP3* are both involved in the inhibition of EMT [20,21]. *COL16A1* encodes the alpha chain of type XVI collagen and plays a role in maintaining the integrity of the extracellular matrix [22]. SDC1 mediates cell binding, cell signaling and cytoskeletal organizations, and it can have a different impact according to cancer type. For instance, in gastric and colorectal cancer a reduced expression of *SDC1* is associated with advanced stages, whereas in pancreatic and breast cancer its overexpression was found to promote cell growth and proliferation [23].

Interestingly, 5 out of 117 genes were deregulated among all groups, thus suggesting that their expression may constitute a gradient from epithelioid to sarcomatoid PM. Again, almost all of them are involved in EMT, which can give intermediate cell states between the epithelial and mesenchymal ones [24].

*SERPINE1* was up-regulated in sarcomatoid in comparison to both epithelioid and biphasic, and in biphasic in comparison to epithelioid PM. This protein belongs to the serine proteinase inhibitor (serpin) superfamily; it is the main inhibitor of tissue plasminogen activator, essential for extracellular matrix remodeling, and urokinase. SERPINE1 can induce epithelial to mesenchimal transition and the acquisition of stem cell properties, thus promoting the transition from a proliferative to an invasive tumor phenotype. *SERPINE1* expression is correlated also to the activation of hypoxia related factors; it is able to promote tumor cell migration and metastasis, and it is usually associated with a poor prognosis [25,26,27].

*CFB*, *MSLN* and *CLDN15* were all up-regulated in epithelioid compared to sarcomatoid and biphasic PM, and in biphasic in comparison to sarcomatoid PM. *CLDN15* and *MSLN* are well known epithelial markers expressed in epithelioid PM. CLDN15 is an integral membrane protein; it is a component of tight junctions and plays a crucial role in maintaining cell polarity and signal transductions [28,29]. It has already been reported as the most significantly up regulated gene in epithelioid in comparison to other PM histotypes [11]; indeed, it is usually suppressed in cells undergoing EMT [30]. *MSLN* gene encodes a cell-surface glicoprotein, expressed at low levels on normal mesothelial cells. MSLN can promote tumor invasion and malignant transformation, and it is highly expressed in epithelioid and biphasic PM, but not in sarcomatoid histotype [31,32,33]. To date, soluble mesothelin is the only tumor biomarker to receive US Food and Drug Administration approval for clinical use in mesothelioma, and it is under evaluation also as therapeutic target [31,33,34]. *CFB* encodes the complement factor B (CFB) involved in the alternative pathway of complement activation. Complement-related proteins are involved in cell-cell and cell-stroma interactions [35] and influence tumor microenvironment and tumor progression. Shimazaki et al. found a correlation between the expression of stromal *CFB* and an enrichment in immunosuppressive regulatory T-cells, myeloid-derived suppressor cells and tumor-associated macrophages in pancreatic ductal adenocarcinoma [35]. A low *CFB* expression has been associated with a poor overall survival and a more aggressive disease in different solid tumors, including lung adenocarcinoma [36] and pancreatic ductal adenocarcinoma [35]. In this study a *CFB* low expression was found in sarcomatoid PM, the most aggressive histotype.

*PAK4* was up-regulated in biphasic PM in comparison to both sarcomatoid and epithelioid histotypes and in epithelioid in comparison to sarcomatoid PM. It encodes a serine threonine kinase with a pro-oncogenic function when overexpressed in human cells. PAK4 is involved in carcinogenesis by promoting cell proliferation, invasion, migration and anti-apoptotic activity. It has a critical role in actin assembly and in the regulation of cytoskeleton dynamics [37]. It has been demonstrated that tumors overexpressing *PAK4* have an activated Wnt/b-catetin signaling pathway, and activated PAK4 promotes anchorage independent cell-growth [38,39]. In addition, PAK4 is involved in the regulation of the anti-tumor immune response and is usually associated with a lack of response to PD-1 blockade. Interestingly, *PAK4* and *PD-L1* over-expression is mediated by the same signaling pathways (mainly PI3K/AKT, and Wnt/β-catenin), and PAK4 inhibition can improve response to immunotherapy [38,39].

The discrimination between biphasic and the other PM histotypes is not always feasible, particularly on small biopsies [10], and the identification of a spindle cell component as malignant can be difficult [8,18]. In this context, the up-mentioned genes can work as diagnostic biormarkes for PM subtyping.

In addition, it has already been demonstrated that gene expression profiles are able to improve also prognostic definition, and that biphasic PM cannot be considered a unique entity, from a clinical nor from a biological point of view [11,12].

The non-negative matrix factorization algorithm identified two clusters of biphasic PM on the basis of gene expression. These two groups had a different percentage of epithelioid component and express differently three genes, namely *ADCY4*, *COL1A1* and *COL4A2*, whose up-regulation is associated with poor survival of PM patients as showed by TCGA data analysis. In our study, all these genes were up-regulated in the cluster of biphasic PM with a lower percentage of epithelioid component, which is usually associated with a worse prognosis [6,8]. From a biological point of view, COL1A1 and COL4A2 have a key role in the organization of extracellular structure, angiogenesis, metallopeptidase activity and EMT promotion [40,41]. Recently, it has been proved, in a murine model, that inhibition of collagen production can delay malignant mesothelioma tumor growth [42]. In addition, *COL1A1* has already been described as a potential prognostic marker for mesothelioma, with a high expression correlated with a poorer overall survival [41]. Furthermore, Zhang and collaborators found that in PM tissues *COL1A1* expression is negatively and positively correlated with neutrophil and macrophages infiltration, respectively. High levels of macrophages infiltration ere indicative of a worse clinical outcome for PM patients [41].

*ADCY4* is a member of the family of adenylate cyclases responsible for the formation of the secondary messenger cyclic adenosine (cAMP). *ADCY4* expression levels have been correlated with overall survival in some cancers, such as breast cancer and in lung adenocarcinoma [43,44]. *ADCY4* has also a role in inflammation: cAMP is involved in the regulation of caspase 11 inflammasome activation and this impacts on perpetuation or resolution of inflammation itself [43]. For these reasons, *ADCY4*, *COL1A1* and *COL4A2* can be valuable candidate as prognostic markers for biphasic PM.

In this study the sample size is limited, but it is relatively high considering the rarity of these tumors. In addition, it was not possible to perform survival analysis on our retrospective cohort because clinical data were not available; TCGA cases include mostly epithelioid PM, with few biphasic and sarcomatoid cases. Consequently, a prospective validation on biphasic PM should be performed. Nevertheless, the biological role, previous reports and the impact on survival of PM patients support our findings.

Although gene expression analysis by the nanoString system proved to be suitable for FFPE samples, it requires specific equipment and expertise. In this context, a prospective validation of identified biomarkers using also more common techniques, such as immunohistochemistry, is warranted both to confirm obtained results and to favor their eventual introduction in clinical practice.

In conclusion, we identified potential diagnostic markers, such as *CTNNA1*, *TIMP3*, *COL16A1*, *SDC1*, *CFB*, *MSLN*, *CLDN15*, *SERPINE1*, and *PAK4*, which could be helpful in the discrimination of biphasic PM from both epithelioid and sarcomatoid subtypes. We propose also *ADCY4*, *COL1A1* and *COL4A2* as potential prognostic markers in biphasic PM and probably in all PM irrespective of the histotype. The validation of these markers could offer additional tools to improve the management of this lethal and orphan disease.

## Data Availability

The data presented in this study are contained within the article or Supplementary Material.

## Supplementary Materials

The following supporting information can be downloaded at: https://www.mdpi.com/article/10.3390/diagnostics12030674/s1. Figure S1: Volcano plot. Biphasic versus epithelioid mesothelioma; Figure S2: Volcano plot. Sarcomatoid versus biphasic mesothelioma; Figure S3: Volcano plot. Sarcomatoid versus epithelioid mesothelioma; Figure S4: Non-negative matrix factorization algorithm performance using the 117 genes compared to a random matrix; Figure S5: Consensus plot. Best fitting clustering by the non-negative matrix factorization algorithm. Table S1: Differentially expressed genes between biphasic and epithelioid mesothelioma; Table S2: Differentially expressed genes between sarcomatoid and biphasic mesothelioma; Table S3: Differentially expressed genes between sarcomatoid and epithelioid mesothelioma.
