# Supplementary material for: Gene Expression Analysis of Biphasic Pleural Mesothelioma: New Potential Diagnostic and Prognostic Markers
Source: Diagnostics (Basel). 2022 Mar 10;12(3):674. doi: 10.3390/diagnostics12030674 (PMC8947498; doi:10.3390/diagnostics12030674)
Supplement: Supplementary file 1 [file diagnostics-12-00674-s001.zip › diagnostics-1605408-supplementary.pdf]

Supplementary Table S1. Biphasic vs Epithelioid						
**Gene**	**logFC**	**AveExpr**	**t**	**P.Value**	**adj.P.Val**	**B**
ITGA5	1.136786283	10.4986036	6.637279408	4.54593E-09	3.66414E-07	10.4966588
COL4A2	1.655301821	11.31559174	6.561890778	6.26349E-09	3.66414E-07	10.18590504
MMP1	2.882160059	2.944917449	4.92499476	4.95272E-06	0.000193156	3.746932275
CFB	-1.905586447	11.06411397	-4.78160045	8.57481E-06	0.000250813	3.221467028
MMP14	0.712142287	11.63962539	4.215419627	6.93279E-05	0.001551833	1.2308087
LAMC1	0.631688399	11.47084275	4.144776373	8.91332E-05	0.001551833	0.992795653
FN1	1.67113331	14.21571314	4.133247097	9.28447E-05	0.001551833	0.954186101
MKI67	1.009607564	8.128595879	4.041269638	0.000128277	0.001867652	0.648579102
BUB1	0.855448316	7.671569339	4.008762677	0.000143666	0.001867652	0.541610178
GLI1	3.030380287	3.440963586	3.935973376	0.000184815	0.002162334	0.304098
COL1A1	1.451430458	15.31475368	3.799307458	0.000294487	0.003132271	-0.13413506
DNMT1	0.462037608	9.183729568	3.726467635	0.00037604	0.003666386	-0.363471354
XPOT	0.372319989	9.761265631	3.628220734	0.000520642	0.004586218	-0.667993364
NOTCH1	0.588902341	8.527145229	3.612182314	0.000548778	0.004586218	-0.717171253
BIRC5	0.904865633	7.781458082	3.583143209	0.000603435	0.004706794	-0.805825757
CENPF	0.844846893	8.678574111	3.491231295	0.000812518	0.00594154	-1.083098475
BMP1	0.687415228	10.44075684	3.462915843	0.000889675	0.006123056	-1.167484589
MSLN	-1.955347881	11.48283101	-3.433389673	0.00097748	0.006353622	-1.254953009
MMP9	1.889185289	7.548592179	3.292149521	0.001522948	0.009378151	-1.665799557
CAV1	0.91135976	9.707321651	3.265407254	0.001654197	0.00967705	-1.742154832
NDC80	0.925477561	6.780240105	3.125159977	0.002534425	0.014120367	-2.134912173
CLDN15	-1.300086789	9.083351443	-3.058224012	0.003093924	0.016454051	-2.317724259
COL16A1	1.080160514	10.48206844	3.026339179	0.003399058	0.017290859	-2.403731172
CDK1	0.80408702	8.358437408	2.970059628	0.004006783	0.018786862	-2.553826129
TUBB2B	2.263501926	6.845698874	2.969415619	0.004014287	0.018786862	-2.555530911
SDC1	0.875394183	7.791323355	2.890529562	0.005038278	0.022672249	-2.762145875
PAK4	0.392790799	8.515931287	2.862332272	0.00545932	0.023657051	-2.834927615
JAG1	0.720825537	8.502440223	2.841348078	0.00579343	0.024208259	-2.888721361
PLK1	0.685893565	7.492734045	2.81624114	0.006217854	0.025085827	-2.952666863
ADCY4	0.790524058	6.687293835	2.775577043	0.006966226	0.026699767	-3.055265078
SERPINE1	1.056381209	11.77478613	2.770039262	0.007074297	0.026699767	-3.069143953
IFITM1	-0.751009907	11.98207007	-2.748988349	0.00749927	0.027419207	-3.121696939
TOP2A	0.625169667	8.766838633	2.702846024	0.008513623	0.030184663	-3.235747376
CCNB1	0.570825205	9.388889576	2.551211748	0.012789949	0.044012472	-3.599315857
PDGFRB	0.565959346	10.6634619	2.456132304	0.016378625	0.054651493	-3.818320628
LAMA3	-1.485593322	7.175297638	-2.445862699	0.016815844	0.054651493	-3.84155479
PLK2	0.541535874	10.92067259	2.425105382	0.017731612	0.055393286	-3.888264025
UBE2T	0.584873739	6.899694973	2.419400684	0.017990982	0.055393286	-3.901041705
MMP7	1.363055293	2.135565251	2.40324238	0.018744132	0.056232397	-3.937094679
CDK7	-0.320860834	8.344222033	-2.279495844	0.025506896	0.074607671	-4.206314362
PTGS2	-1.231695213	5.830247234	-2.22917915	0.028822267	0.082248908	-4.31225573
CD44	0.461153133	11.58540512	2.03306651	0.04561533	0.127071276	-4.705273077
MICAL2	0.537242007	11.12087627	2.001321647	0.049006716	0.133343855	-4.765865174
KRT5	-1.494765238	8.958182028	-1.849238125	0.068397482	0.181875122	-5.044210091
FANCI	0.327161852	8.29957897	1.817377809	0.073187635	0.190287851	-5.099990075
EGFR	-0.458590078	9.713899999	-1.711391757	0.091176987	0.231906683	-5.279145422
TERT	0.391528889	0.488582574	1.681267785	0.096906476	0.241235269	-5.328255004
TNPO2	0.320851583	7.369885649	1.65291994	0.102563211	0.249997827	-5.373731339

EGR3	-0.900482386	7.496076965	-1.595315466	0.114883103	0.272598279	-5.463926782
TIMP3	0.527297002	13.0182603	1.588151862	0.116494991	0.272598279	-5.47493492
CXADR	-0.779909756	8.642778116	-1.577855035	0.118843584	0.272641162	-5.490676777
DSP	-0.977475141	9.655269422	-1.559773022	0.12305939	0.276883628	-5.518089057
CDH1	-1.105470057	7.808435237	-1.501535005	0.137451626	0.303430948	-5.604364145
PKM	0.157611036	12.63528886	1.486660328	0.141332231	0.304311827	-5.625905277
TACC1	0.287311465	10.54961699	1.480168485	0.143052568	0.304311827	-5.635243319
MCM4	0.242701446	9.404836199	1.460282051	0.1484246	0.310101396	-5.663608784
THBS2	0.532102605	12.89264235	1.449242585	0.151473891	0.310920093	-5.679198929
DNMT3A	0.280083198	9.321755553	1.433384538	0.155939075	0.314566755	-5.701398399
ASS1	-0.671633101	10.47032272	-1.419559942	0.15991424	0.31711807	-5.72056282
MMP3	1.053829715	3.822979683	1.334465714	0.186123048	0.362939944	-5.834642639
EIF4G1	-0.13065931	10.47882797	-1.303773202	0.196332518	0.376572207	-5.874142817
CCNB2	0.32643972	7.950533603	1.291009308	0.200699414	0.378739217	-5.890311219
AURKA	0.222266995	6.555570224	1.204415395	0.232248407	0.43131847	-5.995977826
PDCD1	-0.564248776	4.349461681	-1.181166382	0.241300432	0.441127352	-6.023148053
CTNNA1	0.113543001	11.75487714	1.146643587	0.255205001	0.450424342	-6.062552299
MAD2L1	0.270721931	7.097366387	1.144701394	0.256003804	0.450424342	-6.064735629
SOD1	-0.188314386	7.548615996	-1.140022855	0.257935307	0.450424342	-6.069980376
PIK3CA	0.162090012	8.726653237	1.10029556	0.274752364	0.472735686	-6.113679188
PTGIS	-0.569115179	11.38184852	-1.088445914	0.279913234	0.474635484	-6.126423186
LGALS3BP	-0.189188615	12.67530151	-1.074201838	0.286205411	0.478371902	-6.141565472
CDH11	0.248155959	11.81941077	1.058989452	0.293032248	0.482884126	-6.157523641
PECAM1	0.217863236	10.03712172	1.032010691	0.305411814	0.496294197	-6.185281847
ADAMTS8	0.656030425	2.858601785	1.012162846	0.314742165	0.498633897	-6.205258822
NMU	-0.674147574	8.273476115	-1.004207427	0.318535084	0.498633897	-6.2131601
NME2	-0.108178293	13.24187553	-0.998824734	0.321118653	0.498633897	-6.218471757
SFRP1	-0.64828999	7.473634028	-0.993064332	0.323898942	0.498633897	-6.224125361
GNAQ	0.166668376	10.08021556	0.968225925	0.336070031	0.510651865	-6.248138512
ACSL1	-0.195061518	9.86875412	-0.956940017	0.341698231	0.512547346	-6.258853564
CHEK1	0.245634567	7.681722791	0.897226228	0.37249377	0.551667988	-6.313504893
GLI2	0.303285394	8.485835671	0.882477761	0.380362237	0.556279772	-6.326472903
SELE	0.478095907	3.029284033	0.854455412	0.395596985	0.559287712	-6.350532371
CD274	0.430914186	5.788318862	0.845453162	0.400569963	0.559287712	-6.35810005
FGF2	-0.3871562	8.258258681	-0.844352949	0.401180355	0.559287712	-6.359019546
HEG1	-0.198108264	11.9054244	-0.843705368	0.401539895	0.559287712	-6.359560209
SDHB	-0.101325182	9.464685907	-0.817183929	0.416433714	0.57320876	-6.381353124
BAP1	0.194177422	9.805883048	0.795912161	0.428616466	0.583117751	-6.398338356
MYH11	0.500471455	5.064063173	0.781669211	0.436890639	0.586322179	-6.409465051
ITGA7	-0.369395104	5.998539687	-0.774663871	0.440994459	0.586322179	-6.414865181
TGFBR2	0.153678968	10.89551059	0.748911451	0.456272917	0.599819452	-6.434305431
MMP12	0.519366671	1.811860433	0.739834312	0.461729781	0.600248716	-6.44100342
ITGB4	-0.351869172	10.83303003	-0.659239596	0.511778229	0.655967521	-6.49693972
CDKN2A	-0.40163958	2.851197115	-0.652947803	0.515803521	0.655967521	-6.501038673
MAGED1	0.106974735	12.00103811	0.628832838	0.531385364	0.661880218	-6.516389028
SMARCA4	-0.090279576	9.82039815	-0.628246843	0.531767013	0.661880218	-6.516754933
LGALS3	0.110570679	10.76443538	0.609694157	0.543922892	0.669883983	-6.528164993
EMX2	0.358699222	2.795648717	0.600709476	0.549860012	0.67014189	-6.533568974
MMP10	0.18526284	0.644661882	0.580786225	0.563140548	0.679252001	-6.545268675
CCNO	-0.308280572	2.809380922	-0.545517595	0.587030193	0.700842169	-6.565020769

PAPPA	0.327604378	7.595326187	0.531974106	0.596329674	0.704753251	-6.572279718
PPARA	-0.079688532	7.485655812	-0.499024975	0.619234833	0.72185326	-6.589183146
ESR2	-0.294442923	2.265460731	-0.486073475	0.628344352	0.72185326	-6.59553368
CDK4	-0.056705606	9.545011547	-0.484708289	0.62930797	0.72185326	-6.596193409
ITGA3	0.148930461	11.63712073	0.463695074	0.644220631	0.731784601	-6.606115437
PCNA	0.065892358	8.603902257	0.406452746	0.685577322	0.771274487	-6.630925693
GALNT7	-0.078039477	8.412532091	-0.337702713	0.73653865	0.813882222	-6.656426617
SULF1	-0.14345061	11.92354602	-0.336604142	0.73736338	0.813882222	-6.656795985
JUNB	-0.072649627	10.75642772	-0.195066138	0.84587314	0.917388246	-6.694337615
VEGFA	0.048113662	11.32816869	0.193852502	0.846819919	0.917388246	-6.694573212
TPPP	-0.096318693	3.334207197	-0.153774592	0.878204007	0.923240899	-6.701527987
CDKN2B	-0.09423508	6.299283767	-0.150649915	0.880659775	0.923240899	-6.702002878
EEF2	-0.020393666	12.68074024	-0.141726464	0.887679322	0.923240899	-6.703305427
NF2	-0.03176001	8.569145405	-0.138348143	0.890339225	0.923240899	-6.703777821
VWF	-0.03371646	9.406849304	-0.136649516	0.891677108	0.923240899	-6.704011038
RAD21	-0.019345285	12.11611324	-0.113604123	0.90985776	0.933801386	-6.706890536
ITGA4	-0.011578005	8.360768646	-0.048580541	0.961383816	0.978103535	-6.71215654
ITGAM	-0.005282556	7.977981617	-0.017445958	0.986127568	0.994628668	-6.713183135
MCM2	-0.000285092	8.076247361	-0.001387391	0.998896738	0.998896738	-6.713334171

Supplementary Table S2. Sarcomatoid vs Biphasic						
Gene	logFC	AveExpr	t	P.Value	adj.P.Val	B
PAK4	-1.052116623	8.515931287	-6.620443551	4.88357E-09	5.71378E-07	10.42810438
CLDN15	-3.165304622	9.083351443	-6.429495904	1.09744E-08	6.42E-07	9.643366184
KRT5	-5.883618817	8.958182028	-6.285340943	2.01461E-08	7.85698E-07	9.055034478
CXADR	-3.301985982	8.642778116	-5.768493873	1.72199E-07	5.03681E-06	6.980390496
NMU	-4.356972424	8.273476115	-5.604252936	3.36345E-07	7.87048E-06	6.334419833
Gli2	-2.136133739	8.485835671	-5.367167854	8.73318E-07	1.70297E-05	5.415189824
CDH1	-4.529495887	7.808435237	-5.312547311	1.08563E-06	1.81455E-05	5.20580008
NME2	-0.524234162	13.24187553	-4.179640686	7.87594E-05	0.001151857	1.111843584
EGFR	-1.212332254	9.713899999	-3.906707576	0.000204363	0.002656716	0.211370761
CFB	-1.778814791	11.06411397	-3.854249491	0.000244462	0.002860206	0.042800405
DNMT3A	-0.842044483	9.321755553	-3.721132693	0.000382792	0.004071515	-0.378125638
PTGIS	-2.185321419	11.38184852	-3.608996582	0.000554536	0.005406729	-0.724866374
CDH11	-0.960927497	11.81941077	-3.540968066	0.000692094	0.005959892	-0.931615596
SERPINE1	1.5597503	11.77478613	3.531708079	0.000713149	0.005959892	-0.959543783
DSP	-2.542615926	9.655269422	-3.503490219	0.000781118	0.006092717	-1.044329129
SFRP1	-2.595013969	7.473634028	-3.432513318	0.000980208	0.007167767	-1.255439635
ITGB4	-2.087510883	10.83303003	-3.377188303	0.00116769	0.008036455	-1.417829626
CD274	1.907957207	5.788318862	3.232450285	0.001830557	0.011898621	-1.833464441
Gli1	-2.8277687	3.440963586	-3.171490146	0.002204094	0.013032857	-2.004442785
PIK3CA	-0.540456543	8.726653237	-3.167950218	0.002227839	0.013032857	-2.014295835
RAD21	-0.618520057	12.11611324	-3.136441568	0.00244998	0.013649889	-2.101628542
LAMC1	-0.529056545	11.47084275	-2.997538417	0.003698475	0.018911228	-2.478611147
ADAMTS8	-2.248630824	2.858601785	-2.995773902	0.003717592	0.018911228	-2.483314781
JAG1	-0.850487398	8.502440223	-2.894853711	0.004976424	0.024260066	-2.748706681
CTNNA1	-0.327434655	11.75487714	-2.855335768	0.005568694	0.026061486	-2.850662442
ADCY4	-0.892085693	6.687293835	-2.704638546	0.008471989	0.038123951	-3.229078919
EMX2	-1.831680264	2.795648717	-2.648793965	0.009860114	0.042727163	-3.365056564
TIMP3	-1.008988464	13.0182603	-2.624142351	0.010536243	0.044026442	-3.424337325
SDHB	-0.371030094	9.464685907	-2.583901922	0.011730763	0.046030778	-3.520117452
MSLN	-1.702648097	11.48283101	-2.581596848	0.011802764	0.046030778	-3.525566712
LAMA3	-1.804992829	7.175297638	-2.566091155	0.012297575	0.046297951	-3.56211723
ASS1	-1.39992909	10.47032272	-2.555007301	0.012662687	0.046297951	-3.588131773
CCNO	-1.651875196	2.809380922	-2.524086896	0.013733543	0.047479989	-3.660205502
VWF	-0.72072127	9.406849304	-2.52230689	0.013797604	0.047479989	-3.664332189
MMP3	-2.262611174	3.822979683	-2.474066511	0.01563947	0.052280515	-3.775236474
GNAQ	-0.48752532	10.08021556	-2.445598406	0.016827233	0.054688507	-3.839834693
ACSL1	-0.569616812	9.86875412	-2.413016984	0.018285238	0.056499446	-3.912987011
MYH11	-1.788136001	5.064063173	-2.411619041	0.018350247	0.056499446	-3.916106999
HEG1	-0.650971278	11.9054244	-2.393946893	0.019190039	0.057570116	-3.955415389
COL16A1	-0.96420973	10.48206844	-2.332734306	0.022369945	0.06543209	-4.089654791
LGALS3BP	-0.471687652	12.67530151	-2.312649747	0.023510774	0.06709172	-4.133048138
TPPP	-1.603876633	3.334207197	-2.211105315	0.030102439	0.083856794	-4.34744887
PPARA	-0.401802851	7.485655812	-2.17272084	0.032987609	0.088933405	-4.426302847
EEF2	-0.361093894	12.68074024	-2.16690697	0.033445041	0.088933405	-4.438140663
MMP9	1.358089584	7.548592179	2.043609338	0.044534563	0.115789863	-4.682577167
ESR2	-1.406869032	2.265460731	-2.005482775	0.04855022	0.123486429	-4.755581453
CAV1	0.635109654	9.707321651	1.964991526	0.05315071	0.132311341	-4.831763567
TNPO2	-0.398468121	7.369885649	-1.772579318	0.08039384	0.195959985	-5.174498794

PECAM1	-0.408402828	10.03712172	-1.670526811	0.099019117	0.236433402	-5.343134487
EIF4G1	-0.191481416	10.47882797	-1.649881613	0.103185091	0.240748143	-5.376124601
TGFBR2	-0.389727657	10.89551059	-1.639991983	0.105230493	0.240748143	-5.391792888
SOD1	-0.312111568	7.548615996	-1.631565029	0.106999175	0.240748143	-5.40507476
CCNB1	0.419248121	9.388889576	1.618001623	0.109896307	0.242601281	-5.426318619
SELE	-1.033743998	3.029284033	-1.595334819	0.114878772	0.244387371	-5.461452041
CDKN2B	-1.155649162	6.299283767	-1.595316139	0.114882952	0.244387371	-5.461480805
PTGS2	-1.009235809	5.830247234	-1.577243818	0.118984178	0.248591943	-5.489161126
PLK1	0.441705034	7.492734045	1.566065498	0.121578984	0.249556862	-5.506134811
AURKA	0.324508269	6.555570224	1.518418727	0.133149004	0.26859368	-5.577215452
BUB1	0.362681525	7.671569339	1.467595822	0.146430895	0.290379911	-5.650759149
NDC80	0.484563591	6.780240105	1.41293311	0.161847249	0.315602135	-5.727221582
LGALS3	-0.292320143	10.76443538	-1.391859059	0.16811412	0.322448394	-5.755966304
TUBB2B	1.212161283	6.845698874	1.373140549	0.173834908	0.324611579	-5.781154435
SMARCA4	-0.226729726	9.82039815	-1.362428541	0.177174834	0.324611579	-5.79542307
PDGFRB	0.36297373	10.6634619	1.360210674	0.177872404	0.324611579	-5.798364046
TACC1	-0.3040104	10.54961699	-1.352418494	0.180339766	0.324611579	-5.808660651
CDK1	0.415797683	8.358437408	1.326198845	0.188832696	0.33474887	-5.842893771
CENPF	0.357629367	8.678574111	1.276139782	0.205877428	0.359517299	-5.906476053
ITGA3	-0.44733062	11.63712073	-1.202657706	0.232924053	0.397159902	-5.995563861
EGR3	-0.783950135	7.496076965	-1.199290121	0.234222506	0.397159902	-5.999525094
TERT	-0.31321848	0.488582574	-1.161408287	0.249190228	0.416503666	-6.043348221
CDK7	-0.185901147	8.344222033	-1.140429993	0.257766814	0.420004591	-6.067033448
CDK4	-0.153603224	9.545011547	-1.133755073	0.260539045	0.420004591	-6.074482299
JUNB	-0.485550405	10.75642772	-1.125763797	0.263885581	0.420004591	-6.083344596
FGF2	0.592257759	8.258258681	1.11535477	0.268289807	0.420004591	-6.094797345
ITGA5	0.220600308	10.4986036	1.112197345	0.269635892	0.420004591	-6.098251042
PDCD1	0.611173683	4.349461681	1.104764118	0.272823495	0.420004591	-6.106344372
MKI67	0.295758664	8.128595879	1.022273663	0.309965495	0.470986531	-6.192628466
FN1	0.473519196	14.21571314	1.0113054	0.315149509	0.472724263	-6.203611646
UBE2T	0.27548401	6.899694973	0.984025917	0.328293535	0.486206881	-6.230428306
MICAL2	-0.294478104	11.12087627	-0.947249535	0.346579625	0.500832522	-6.265449725
TOP2A	0.250683283	8.766838633	0.935865082	0.352371905	0.500832522	-6.276027153
ITGA7	-0.513212983	5.998539687	-0.929360618	0.355709203	0.500832522	-6.282014472
MAGED1	-0.18255834	12.00103811	-0.926659398	0.357101099	0.500832522	-6.284488943
CHEK1	0.2922791	7.681722791	0.921880656	0.359572067	0.500832522	-6.288849306
VEGFA	0.262411537	11.32816869	0.912957128	0.364215462	0.501331871	-6.296932599
MMP12	0.717787414	1.811860433	0.882918659	0.380125513	0.5171475	-6.323577508
SULF1	-0.412802417	11.92354602	-0.836418733	0.405599061	0.541967292	-6.36310199
BMP1	-0.191445342	10.44075684	-0.832783392	0.407633519	0.541967292	-6.366103635
MAD2L1	0.221961006	7.097366387	0.810419571	0.42028501	0.552509508	-6.384286742
IFITM1	-0.240074969	11.98207007	-0.758819665	0.450358893	0.585466561	-6.42438401
XPOT	-0.088623044	9.761265631	-0.745741776	0.458174201	0.589081115	-6.434134358
GALNT7	-0.194942017	8.412532091	-0.728433843	0.468635734	0.595982401	-6.446781717
PKM	0.085557766	12.63528886	0.696865603	0.488059628	0.6140105	-6.469095302
MMP14	-0.132698146	11.63962539	-0.678271169	0.499704971	0.621973208	-6.481782024
BIRC5	0.192886095	7.781458082	0.659546554	0.511582276	0.630053961	-6.494215124
COL4A2	-0.182980272	11.31559174	-0.626354984	0.533000115	0.649593891	-6.515408719
NOTCH1	-0.114789418	8.527145229	-0.60798477	0.545049953	0.657431387	-6.526673186
MMP7	0.376420339	2.135565251	0.573088436	0.568313733	0.678497007	-6.547156806

MCM4	0.104949652	9.404836199	0.545267799	0.58720109	0.693964925	-6.56262753
CCNB2	0.151774301	7.950533603	0.518309396	0.605781192	0.708763995	-6.576890271
DNMT1	0.066280102	9.183729568	0.461601972	0.645714231	0.748005594	-6.604547919
MMP1	0.294032724	2.944917449	0.433858198	0.665647507	0.757796653	-6.61691986
FANCI	-0.090023912	8.29957897	-0.431822141	0.667120131	0.757796653	-6.617797774
SDC1	-0.135113544	7.791323355	-0.385245087	0.701155565	0.78880001	-6.636758377
CDKN2A	0.241821583	2.851197115	0.339470066	0.735212496	0.819236781	-6.653294315
PLK2	0.078947086	10.92067259	0.305283937	0.761002772	0.839974757	-6.664285392
PAPPA	-0.208623102	7.595326187	-0.292528146	0.77069748	0.842725281	-6.668088653
ITGA4	0.075339331	8.360768646	0.272969941	0.785632932	0.851102343	-6.673605608
CD44	0.068758402	11.58540512	0.261756009	0.794233208	0.852525554	-6.676597039
COL1A1	-0.096857243	15.31475368	-0.218929747	0.827304525	0.879951177	-6.686868681
THBS2	-0.065048884	12.89264235	-0.15298541	0.878824135	0.926328142	-6.699109865
NF2	0.035407767	8.569145405	0.133185121	0.894406726	0.934335598	-6.701938572
PCNA	-0.016489152	8.603902257	-0.087828971	0.930248694	0.952671689	-6.706943456
BAP1	-0.024431301	9.805883048	-0.086472391	0.931323311	0.952671689	-6.70706152
MMP10	-0.02958323	0.644661882	-0.080082577	0.936386703	0.952671689	-6.707592915
MCM2	0.015219179	8.076247361	0.063954174	0.949178317	0.957360888	-6.708752886
ITGAM	-0.010108647	7.977981617	-0.028827584	0.977079326	0.977079326	-6.710380297

Supplementary Table S3. Sarcomatoid vs Epithelioid						
**Gene**	**logFC**	**AveExpr**	**t**	**P.Value**	**adj.P.Val**	**B**
CLDN15	-4.465391411	9.083351443	-9.516454056	1.69055E-14	1.97795E-12	22.61994464
CFB	-3.684401238	11.06411397	-8.375875663	2.43498E-12	1.42446E-10	17.71470096
KRT5	-7.378384055	8.958182028	-8.269890894	3.86857E-12	1.50874E-10	17.25811621
CXADR	-4.081895739	8.642778116	-7.481752345	1.19993E-10	3.50978E-09	13.87379479
ITGA5	1.357386591	10.4986036	7.18014979	4.4289E-10	1.03636E-08	12.58883966
CDH1	-5.634965943	7.808435237	-6.934233267	1.277E-09	2.49015E-08	11.54785237
NMU	-5.031119997	8.273476115	-6.789719034	2.37214E-09	3.96487E-08	10.93954672
SERPINE1	2.616131509	11.77478613	6.215033916	2.70578E-08	3.9572E-07	8.552596647
MSLN	-3.657995978	11.48283101	-5.819168849	1.3988E-07	1.81843E-06	6.946293352
EGFR	-1.670922333	9.713899999	-5.649366093	2.80033E-07	3.27638E-06	6.268999979
NME2	-0.632412455	13.24187553	-5.29015326	1.18665E-06	1.16794E-05	4.863279277
COL4A2	1.472321549	11.31559174	5.287777186	1.19789E-06	1.16794E-05	4.854113905
BUB1	1.218129841	7.671569339	5.171646441	1.89545E-06	1.7059E-05	4.408443604
MMP9	3.247274873	7.548592179	5.126756874	2.26082E-06	1.8894E-05	4.237403196
DSP	-3.520091067	9.655269422	-5.088950846	2.62134E-06	2.04465E-05	4.0939004
CAV1	1.546469414	9.707321651	5.020043876	3.42868E-06	2.50723E-05	3.833661435
MMP1	3.176192783	2.944917449	4.917147158	5.10471E-06	3.43404E-05	3.448310858
LAMA3	-3.29058615	7.175297638	-4.908220611	5.28314E-06	3.43404E-05	3.415069201
GLI2	-1.832848346	8.485835671	-4.831672402	7.08508E-06	4.36292E-05	3.131277524
FN1	2.144652506	14.21571314	4.80569143	7.82344E-06	4.57671E-05	3.035480236
PTGIS	-2.754436598	11.38184852	-4.772634088	8.87205E-06	4.943E-05	2.913981315
MKI67	1.305366228	8.128595879	4.733869472	1.02769E-05	5.46542E-05	2.772069965
CENPF	1.20247626	8.678574111	4.501899498	2.44722E-05	0.000119675	1.936091539
SFRP1	-3.243303959	7.473634028	-4.501055766	2.45486E-05	0.000119675	1.933093677
PAK4	-0.659325824	8.515931287	-4.352888084	4.22468E-05	0.000197715	1.411677201
NDC80	1.410041152	6.780240105	4.313768371	4.86824E-05	0.000219071	1.275720444
PLK1	1.127598599	7.492734045	4.194559037	7.46854E-05	0.000323637	0.865982502
CD274	2.338871394	5.788318862	4.157418812	8.52278E-05	0.00035613	0.739761877
ITGB4	-2.439380055	10.83303003	-4.140570725	9.04702E-05	0.000365001	0.68273304
TUBB2B	3.475663209	6.845698874	4.130917112	9.36126E-05	0.000365089	0.650121586
CDK1	1.219884703	8.358437408	4.082249798	0.000111124	0.000419404	0.48644176
CCNB1	0.990073326	9.388889576	4.008938545	0.000143578	0.000524956	0.242199369
ASS1	-2.071562191	10.47032272	-3.966781633	0.000166179	0.000589179	0.10303604
BIRC5	1.097751728	7.781458082	3.938246315	0.000183374	0.000631023	0.009379733
DNMT1	0.52831771	9.183729568	3.860413889	0.000239386	0.000800233	-0.243820266
PTGS2	-2.240931023	5.830247234	-3.674420721	0.000447059	0.001452943	-0.835108164
PDGFRB	0.928933076	10.6634619	3.65232673	0.00048092	0.001520746	-0.904018857
SDHB	-0.472355276	9.464685907	-3.451356885	0.000923122	0.002842243	-1.517367978
TOP2A	0.87585295	8.766838633	3.430625237	0.000986108	0.002958323	-1.579226663
LGALS3BP	-0.660876267	12.67530151	-3.39961489	0.001087944	0.00308838	-1.671249828
ACSL1	-0.76467833	9.86875412	-3.398682328	0.001091155	0.00308838	-1.674007786
RAD21	-0.637865342	12.11611324	-3.393646721	0.001108649	0.00308838	-1.688890552
IFITM1	-0.991084876	11.98207007	-3.286674957	0.001548993	0.004214701	-2.001206834
HEG1	-0.849079542	11.9054244	-3.276085834	0.001600562	0.00425604	-2.031719744
CDK7	-0.506761981	8.344222033	-3.261705878	0.001673178	0.004350262	-2.073038415
UBE2T	0.860357749	6.899694973	3.224359265	0.001876472	0.004772766	-2.179711953
COL1A1	1.354573214	15.31475368	3.212398136	0.00194633	0.00484512	-2.2136813
CCNO	-1.960155768	2.809380922	-3.142475247	0.002405902	0.005864386	-2.410344944

MMP14	0.579444141	11.63962539	3.107450992	0.002672475	0.006381215	-2.507611376
EIF4G1	-0.322140726	10.47882797	-2.912232178	0.004734838	0.011079521	-3.034189999
MMP7	1.739475632	2.135565251	2.778568599	0.006908477	0.015848859	-3.379061749
VWF	-0.75443773	9.406849304	-2.770181326	0.007071506	0.015910888	-3.400266406
CDH11	-0.712771538	11.81941077	-2.755725462	0.007360786	0.016249282	-3.436691459
SOD1	-0.500425954	7.548615996	-2.744660036	0.007589494	0.016443903	-3.464468732
PPARA	-0.481491383	7.485655812	-2.731704047	0.007865526	0.01673212	-3.496876078
EGR3	-1.684432522	7.496076965	-2.703607515	0.008495914	0.017750391	-3.566724421
AURKA	0.546775263	6.555570224	2.684286412	0.008955798	0.018382954	-3.614413376
NOTCH1	0.474112922	8.527145229	2.63467377	0.010242405	0.020661403	-3.73557746
DNMT3A	-0.561961285	9.321755553	-2.605557906	0.011073486	0.021959286	-3.805812711
ESR2	-1.701311955	2.265460731	-2.544505712	0.013017622	0.025384363	-3.950973405
PLK2	0.620482961	10.92067259	2.517398198	0.013975661	0.026805777	-4.01450066
XPOT	0.283696945	9.761265631	2.504670964	0.014447005	0.027262896	-4.044129751
TPPP	-1.700195325	3.334207197	-2.459186673	0.016250569	0.030179627	-4.148979903
EEF2	-0.38148756	12.68074024	-2.40189884	0.018808005	0.034383384	-4.278714677
PIK3CA	-0.378366531	8.726653237	-2.326936291	0.02269413	0.040849434	-4.444516061
BMP1	0.495969887	10.44075684	2.26358481	0.026516926	0.047007278	-4.581093028
EMX2	-1.472981043	2.795648717	-2.234857724	0.028430048	0.049646501	-4.641944009
ADAMTS8	-1.592600399	2.858601785	-2.226137083	0.029034332	0.049956129	-4.660282232
SDC1	0.740280639	7.791323355	2.214566998	0.029853455	0.050621077	-4.684515639
CD44	0.529911535	11.58540512	2.116549696	0.037644134	0.062919482	-4.885353123
PKM	0.243168801	12.63528886	2.078029256	0.041158205	0.067824085	-4.962079682
SMARCA4	-0.317009302	9.82039815	-1.998625782	0.049304423	0.080119688	-5.116271012
CTNNA1	-0.213891655	11.75487714	-1.956953893	0.054106836	0.086719176	-5.195035873
MCM4	0.347651098	9.404836199	1.895076137	0.061969228	0.097978374	-5.309226851
MAD2L1	0.492682936	7.097366387	1.887360963	0.063014081	0.098301966	-5.323231585
MYH11	-1.287664546	5.064063173	-1.822070202	0.072465025	0.111557998	-5.439665401
CDKN2B	-1.249884241	6.299283767	-1.810275831	0.074292745	0.112886379	-5.46029942
CHEK1	0.537913666	7.681722791	1.780097253	0.079144981	0.118717471	-5.512537388
CCNB2	0.478214021	7.950533603	1.713433904	0.090798849	0.134474245	-5.625066017
GNAQ	-0.320856944	10.08021556	-1.688704341	0.095465437	0.139618201	-5.665801796
ITGA7	-0.882608087	5.998539687	-1.676906096	0.097759899	0.141208743	-5.685043326
CDK4	-0.210308831	9.545011547	-1.628660751	0.107614275	0.153547198	-5.762422592
MMP12	1.237154085	1.811860433	1.59662476	0.114590441	0.161531104	-5.812642475
MMP3	-1.208781459	3.822979683	-1.386766824	0.169655949	0.236306501	-6.11840566
JUNB	-0.558200032	10.75642772	-1.357866348	0.178612014	0.245854184	-6.157324081
TIMP3	-0.481691462	13.0182603	-1.31439031	0.192754526	0.262235809	-6.214400304
FANCI	0.23713794	8.29957897	1.193444644	0.236488778	0.318036633	-6.363825321
SULF1	-0.556253027	11.92354602	-1.182518972	0.240766952	0.320110607	-6.376641859
THBS2	0.467053721	12.89264235	1.152474202	0.25281756	0.332355669	-6.41130076
VEGFA	0.310525199	11.32816869	1.133492154	0.26064867	0.33884327	-6.432754411
GALNT7	-0.272981494	8.412532091	-1.070217572	0.287982739	0.370263521	-6.501778523
TGFBR2	-0.236048689	10.89551059	-1.042164562	0.300711641	0.382426761	-6.531151255
LGALS3	-0.181749463	10.76443538	-0.907954041	0.366835497	0.461502721	-6.661159646
SELE	-0.555648091	3.029284033	-0.899689973	0.371189436	0.462012383	-6.668593845
ITGA3	-0.298400159	11.63712073	-0.841718111	0.402644462	0.495888443	-6.718872367
MICAL2	0.242763904	11.12087627	0.819312755	0.415226073	0.501929897	-6.737424617
PECAM1	-0.190539592	10.03712172	-0.817719116	0.416129915	0.501929897	-6.738725476
BAP1	0.169746121	9.805883048	0.630354623	0.530394913	0.63322658	-6.87426333

LAMC1	0.102631853	11.47084275	0.610097223	0.543657309	0.642504092	-6.886841347
JAG1	-0.129661861	8.502440223	-0.463047133	0.644682834	0.754278916	-6.965928815
MMP10	0.15567961	0.644661882	0.442158929	0.659657468	0.764157661	-6.975416288
FGF2	0.20510156	8.258258681	0.405252258	0.686455594	0.782022797	-6.991114429
MAGED1	-0.075583605	12.00103811	-0.402531868	0.688447419	0.782022797	-6.992217679
TNPO2	-0.077616538	7.369885649	-0.362260159	0.718185084	0.80795822	-7.007684022
ADCY4	-0.101561635	6.687293835	-0.323062503	0.747554566	0.832989374	-7.02117889
TERT	0.078310409	0.488582574	0.304657059	0.761478335	0.840499671	-7.026984371
COL16A1	0.115950784	10.48206844	0.294321281	0.769332404	0.841232629	-7.030095605
PCNA	0.049403206	8.603902257	0.27608882	0.783245664	0.848516136	-7.035322691
ITGA4	0.063761326	8.360768646	0.242384372	0.809149574	0.858583705	-7.044107301
GLI1	0.202611587	3.440963586	0.238417413	0.812213119	0.858583705	-7.045066238
CDKN2A	-0.159817996	2.851197115	-0.235389082	0.814553772	0.858583705	-7.045787644
PAPPA	0.118981276	7.595326187	0.175040318	0.861523665	0.899984543	-7.0582431
PDCD1	0.046924906	4.349461681	0.088994372	0.929325623	0.962222105	-7.069671456
TACC1	-0.016698935	10.54961699	-0.077940942	0.938084367	0.962770797	-7.070599802
MCM2	0.014934087	8.076247361	0.065843142	0.947679399	0.96416078	-7.071474971
ITGAM	-0.015391203	7.977981617	-0.046051309	0.963392798	0.971697909	-7.072589218
NF2	0.003647757	8.569145405	0.014395849	0.988552723	0.988552723	-7.07355199

SUPPLEMENTARY FIGURES

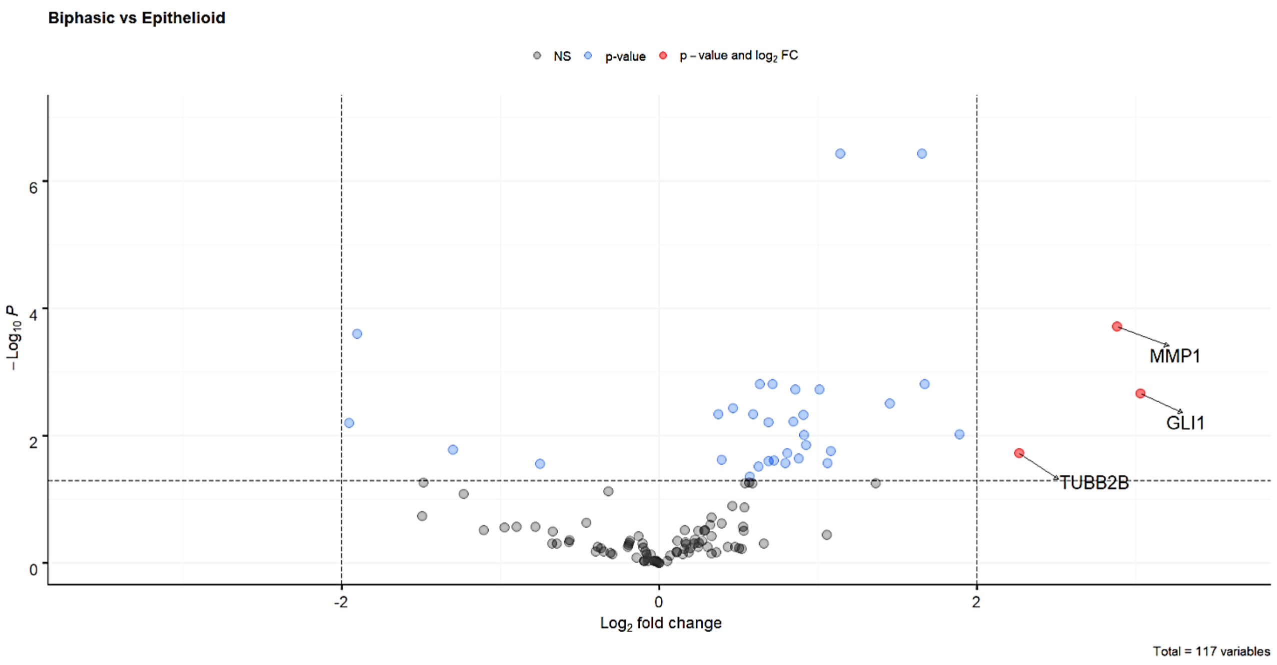

Figure S1: Volcano plot. Biphasic versus epithelioid mesothelioma.

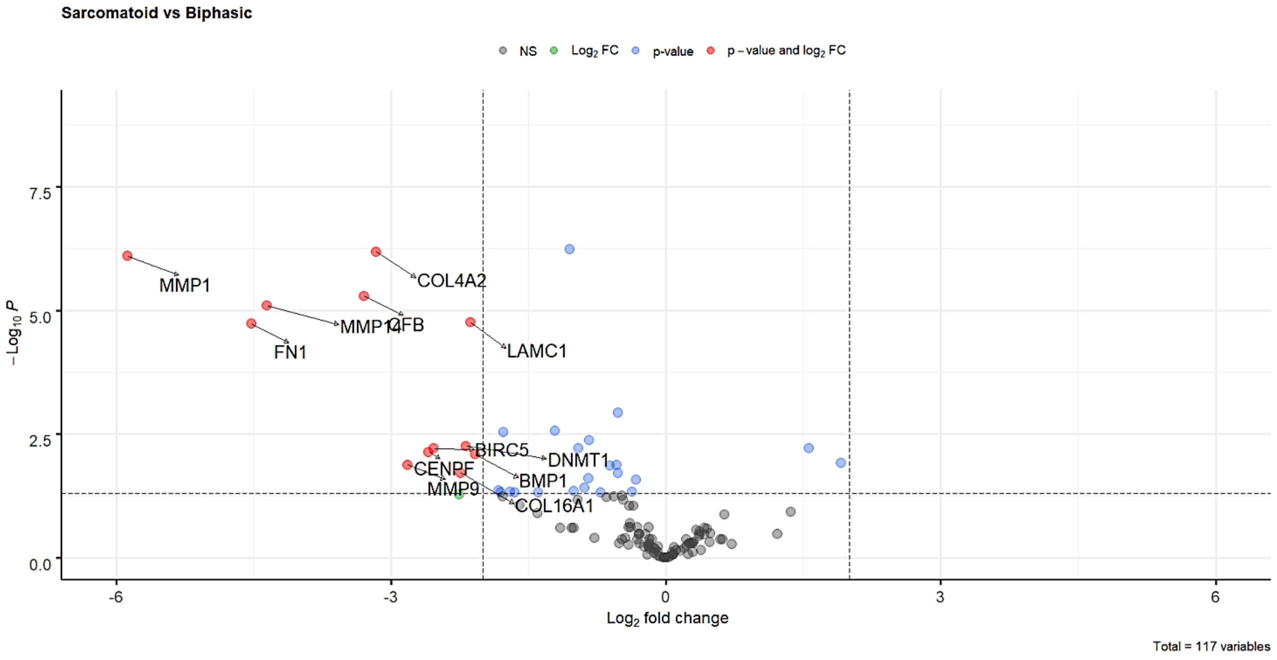

Figure S2: Volcano plot. Sarcomatoid versus biphasic mesothelioma.

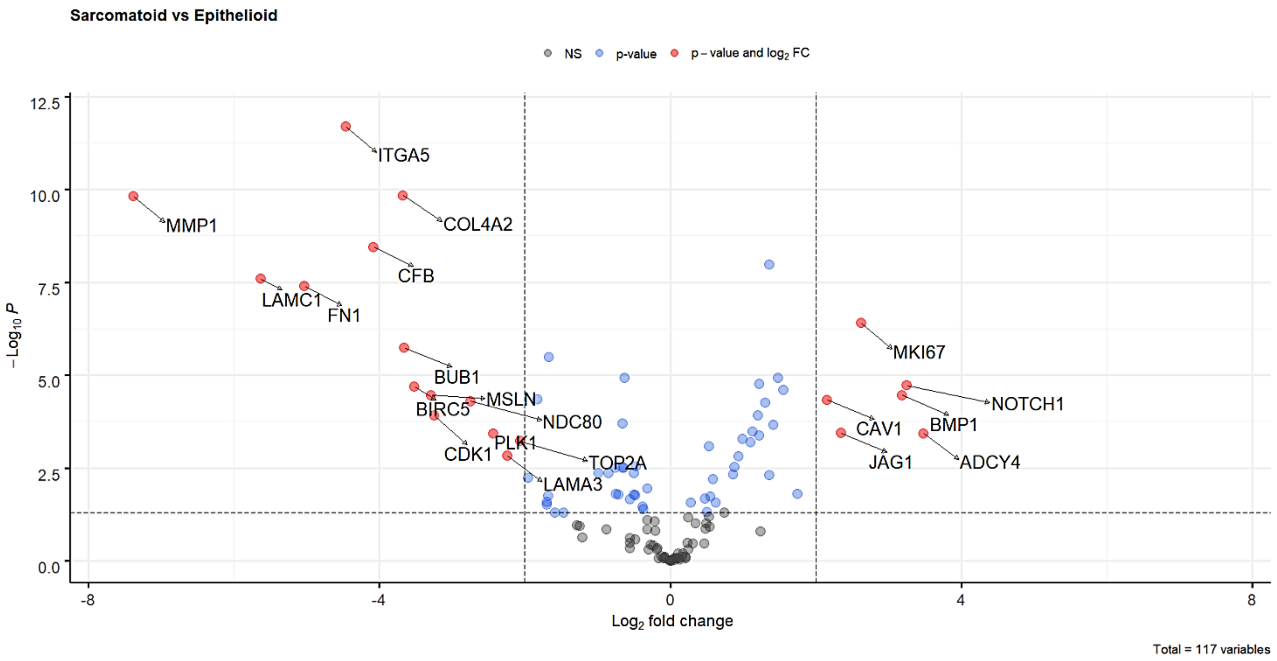

Figure S3: Volcano plot. Sarcomatoid versus epithelioid mesothelioma.

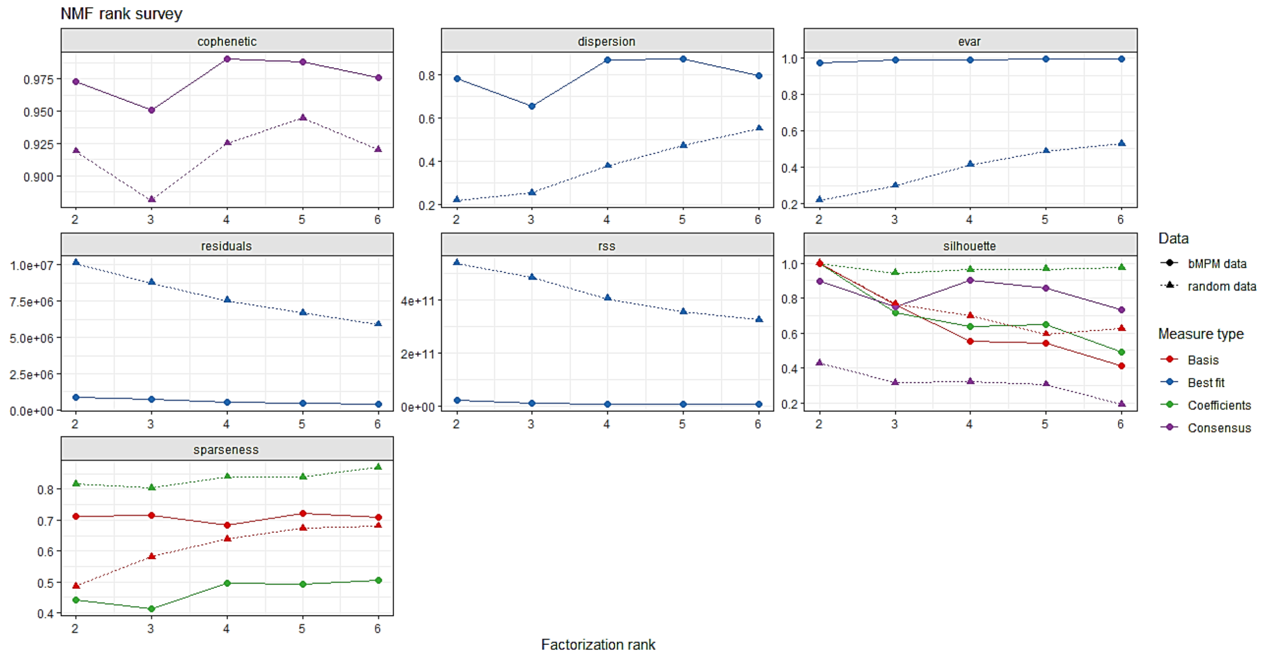

Figure S4: Non-negative matrix factorization algorithm performance using the 117 genes compared to a random matrix.

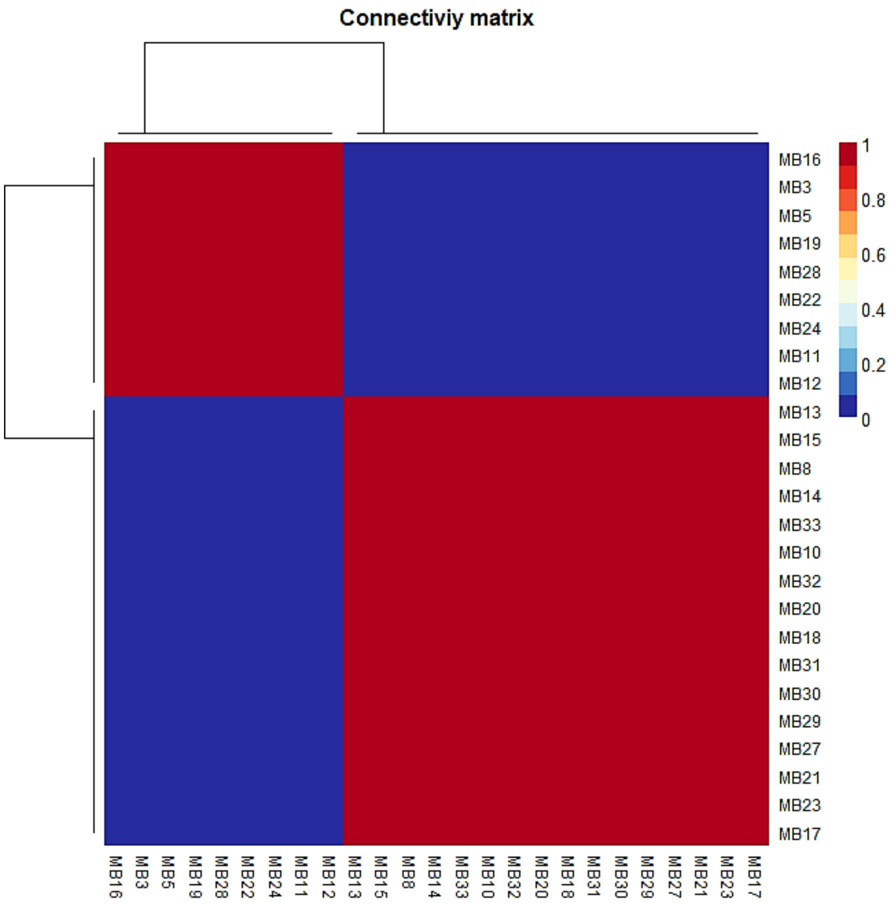

Figure S5: Consensus plot. Best fitting clustering by the non-negative matrix factorization algorithm.
